# Correcting magnification error in foveal avascular zone area measurements of optical coherence tomography angiography images with estimated axial length

**DOI:** 10.1186/s40662-022-00299-x

**Published:** 2022-08-01

**Authors:** Deepaysh D. C. S. Dutt, Seyhan Yazar, Jason Charng, David A. Mackey, Fred K. Chen, Danuta M. Sampson

**Affiliations:** 1 Centre for Ophthalmology and Visual Science (Incorporating Lions Eye Institute), The University of Western Australia, Perth, WA Australia; 2 Garvan Institute of Medical Research, Sydney, Australia; 3 Ophthalmology, Department of Surgery, University of Melbourne, East Melbourne, VIC Australia; 4 Centre for Eye Research Australia, Royal Victorian Eye and Ear Hospital, East Melbourne, VIC Australia; 5 Surrey Biophotonics, Centre for Vision, Speech and Signal Processing and School of Biosciences and Medicine, The University of Surrey, Guildford, UK; 6 Institute of Ophthalmology, University College London, London, UK; 7 Lions Eye Institute, 2 Verdun Street, Nedlands, WA 6009 Australia

**Keywords:** OCTA, Keratometry, Spherical equivalent, Axial length, Littmann-Bennett formula

## Abstract

**Background:**

To generate and validate a method to estimate axial length estimated (AL_est_) from spherical equivalent (SE) and corneal curvature [keratometry (K)], and to determine if this AL_est_ can replace actual axial length (AL_act_) for correcting transverse magnification error in optical coherence tomography angiography (OCTA) images using the Littmann-Bennett formula.

**Methods:**

Data from 1301 participants of the Raine Study Gen2-20 year follow-up were divided into two datasets to generate (n = 650) and validate (n = 651) a relationship between AL, SE, and K. The developed formula was then applied to a separate dataset of 46 participants with AL, SE, and K measurements and OCTA images to estimate and compare the performance of AL_est_ against AL_act_ in correcting transverse magnification error in OCTA images when measuring the foveal avascular zone area (FAZA).

**Results:**

The formula for AL_est_ yielded the equation: AL_est_ = 2.102K − 0.4125SE + 7.268, R^2^ = 0.794. There was good agreement between AL_est_ and AL_act_ for both study cohorts. The mean difference [standard deviation (SD)] between FAZA corrected with AL_est_ and AL_act_ was 0.002 (0.015) mm^2^ with the 95% limits of agreement (LoA) of − 0.027 to 0.031 mm^2^. In comparison, mean difference (SD) between FAZA uncorrected and corrected with AL_act_ was − 0.005 (0.030) mm^2^, with 95% LoA of − 0.064 to 0.054 mm^2^.

**Conclusions:**

AL_act_ is more accurate than AL_est_ and hence should be used preferentially in magnification error correction in the clinical setting. FAZA corrected with AL_est_ is comparable to FAZA corrected with AL_act_, while FAZA measurements using images corrected with AL_est_ have a greater accuracy than measurements on uncorrected images. Hence, in the absence of AL_act_, clinicians should use AL_est_ to correct for magnification error as this provides for more accurate measurements of fundus parameters than uncorrected images.

**Supplementary Information:**

The online version contains supplementary material available at 10.1186/s40662-022-00299-x.

## Background

Quantitative measurements derived from optical coherence tomography angiography (OCTA) of the retinal fundus, including retinal vessel density and foveal avascular zone area (FAZA), can be calculated accurately only if the lateral scale of the fundus image is known [1]. Most ocular imaging instruments define lateral scale by calculating the linear distance on the surface of the retina subtended by a fixed angular field of view using a standardised axial length (AL) [2]. However, this does not account for the differences in AL between participants [3]. Therefore, before quantitative analysis of retinal fundus images can be performed, correction should be undertaken to consider the lateral magnification arising from the deviation of the actual AL from the standardised AL [1, 3–8]. In a systematic review by Llanas et al. of 989 articles, only 8% of 509 studies included appropriate magnification error correction when it was needed [1]. Whilst it is true that various fundus parameters, including vessel density, are impacted by magnification error, FAZA has shown to be highly sensitive to magnification error correction [3]. The FAZA in uncorrected images may deviate from its true size by as much as 51% [3, 8]. This deviation may contribute to errors in diagnosis and monitoring of diabetic retinopathy and other retinal vascular diseases where FAZA serves as a potential biomarker [9–11].

The Littmann-Bennett method is considered the reference standard in correcting image magnification error induced by AL variation [4]. This method has been developed with reference to previous approaches in determining the true size of fundus parameters [5, 12–14]. However, a specialised biometry device is required to measure AL [15]. An alternative approach is needed that would allow for the transverse magnification error of the eye to be corrected in various clinical settings where biometry devices may be unavailable, or if retrospective analysis of OCTA images is needed to be performed in the absence of AL measurements. In their recent paper, Morgan et al. [15] explored the potential of estimating AL from spherical equivalent (SE) and corneal curvature [keratometry (K)], in an effort to develop a simple and cost-effective means for all eyecare practitioners to manage myopia [15]. Morgan et al. [15] have shown that SE and K can be used to provide an estimate of AL. However, they did not investigate whether the estimated AL can be applied in correcting for eye transverse magnification error in fundus imaging parameters.

The aim of this study is to generate and validate a method of AL estimation using SE and K, and to assess the performance of this estimated AL in correcting eye transverse magnification error in FAZA measurements of OCTA images. The FAZA metric was chosen as, compared to other fundus metrics, it has the strongest dependence on image correction [3].

## Methods

### Study participants

Data from 1301 participants of the Raine Study Gen2-20 year follow-up were divided into two separate datasets: 650 participants were used to generate a formula to estimate AL using SE and K measurements (training dataset) and 651 participants were used to validate this estimated AL against actual AL measurements (validation dataset). The Raine Study Gen2-20 year follow-up is a prospective cohort follow-up study of the offspring of 2900 women recruited from Western Australia’s largest maternity hospital. Extensive data were collected during pregnancy and the children were assessed at birth and at ages 1, 2, 3, 5, 8, 10, 14, 17, 18 and 20 years with questionnaire data, physical measurements and biological samples analysing cardiovascular, respiratory, immunological, musculoskeletal, nutritional, psychiatric, neurocognitive, and ophthalmic health. There were no specific inclusion or exclusion criteria for astigmatism for the Raine Study Gen2-20 year follow-up cohort. Further details of the recruitment process and methodology for the Raine Study can be found elsewhere [16, 17]. All participants in the Raine Study Gen2-20 year follow-up underwent ophthalmic examination, including AL measurement with the IOLMaster 500 (IOLMaster; Carl Zeiss Meditec, Inc., Dublin, CA, USA), K measurement with the Oculus Pentacam (OculusOptikgerate GmbH, Wetzlar, Germany) and pre- and post-cycloplegic refraction measurements with the Nidek ARK-510A (Nidek Co, Ltd, Gamagori, Japan) autorefractor. Only data from the right eye were used for each participant.

Data from 46 participants of studies RA/4/20/4275 and RA/4/1/8570 were used to validate the accuracy of the estimated AL in correcting eye transverse magnification error of measured FAZA in OCTA images. RA/4/20/4275 is a retrospective cross-sectional study of the anonymized OCTA images collected as part of routine clinical care. RA/4/1/8570 is a separate cohort of healthy subjects recruited prospectively to establish a normative database. All participants included in this study had no known eye disease and were recruited through voluntary participation. There were no specific inclusion or exclusion criteria for astigmatism for participants of these studies. Participants underwent ophthalmic examination, ocular biometry with the IOLMaster 500, autorefraction (ARK1, Auto Ref/Keratometer; Nidek, Gamagori, Japan), and OCTA imaging (RTVue XR Avanti; Optovue, Inc., Fremont, CA, USA). Only data from the right eye were used for each participant.

### Generation and validation of relationship between AL, SE, and K

A multiple linear regression model was applied to the training dataset to generate a relationship between AL, SE, and K [16]. Post-cycloplegic SE was used in this model, with K defined as the average of the horizontal and vertical meridian of K. The AL estimated using this regression formula containing SE and K is defined as estimated AL (AL_est_).

To evaluate the efficacy of the generated formula, the validation dataset was used to compare values of the actual AL (AL_act_) and AL_est_ using Bland-Altman analysis and intraclass coefficient (ICC). Post-cycloplegic and non-cycloplegic SE measures were used respectively in this dataset to investigate the impact of cycloplegia on AL_est_.

### Fundus image magnification

Two formulae were used to calculate the true linear dimension of fundus measurements. First, the magnification factor of the eye (q) was calculated using the Littmann-Bennet formula as follows: $\mathrm{q}=0.01306\times \left(\mathrm{AL}-1.82\right)$, where AL is the axial length of the eye and 1.82 is a constant related to the distance between the corneal apex and the AL. This method is considered the gold standard in estimating the magnification factor of the eye [4]. The factor q is used to adjust measured linear fundus dimensions to estimate the true value of fundus measurements using the Littmann formula as follows: ${\mathrm{D}}_{\mathrm{t}}=\mathrm{ p}\cdot \mathrm{q}\cdot {\mathrm{D}}_{\mathrm{m}}$, where D_m_ is the measured linear dimension of the image, D_t_ is the true linear dimension on the fundus and p is the magnification factor of the imaging system. The factor p can be accurately calculated using the Bennet formula if the AL at which D_m_ = D_t_ is known, and if the effects arising from image distortion are omitted. The factor $\mathrm{p}$ for RTVue XR Avanti OCTA instrument used in the present study is 3.48 [3], where the defined axial length for the Optovue RTVue XR Avanti system is 23.95 mm (Optovue, Inc., personal communication, 2017).

### Evaluating performance of AL_est_ on magnification factor correction of FAZA

Retinal OCTA images of the superficial vascular plexus of 46 eyes (OCTA test dataset) in the OCTA cohort were used to assess the performance of AL_est_ against AL_act_ in correcting OCTA image magnification error and FAZA measurements. The OCTA images used were of a scan size of 3 × 3 mm. OCTA image quality was assessed by the scan quality index (SQI). The images of SQI ≥ 7 were considered for further analysis, if deemed acceptable by the senior author who visually inspected the images. FAZA measurements were derived from the RTVue XR Avanti system, Optovue software. FAZA correction was performed by multiplying uncorrected FAZA by the square of the linear magnification factor (q). Bland-Altman analysis was performed and intraclass coefficient was calculated to compare AL_est_ and AL_act_ in their performance in calculating q and correcting for magnification error in FAZA measurements.

### Statistical analysis

The performance of the regression formula for AL_est_ based on the training dataset was evaluated by Bland-Altman plots and the 95% limits of agreements (95% LoA = $\overline{\mathrm{d} }$  ± 1.96SD, where LoA is the limits of agreement, $\overline{\mathrm{d} }$ is the mean difference between methods and SD is the standard deviation of difference of both) [18, 19]. The ICC was calculated to assess the absolute agreement between the two measures. Additionally, the t-statistic (t_stat_) was calculated, where ${\mathrm{t}}_{\mathrm{stat}}=\frac{\overline{\mathrm{d}}}{\mathrm{SE }\left(\overline{\mathrm{d} }\right)}$ and $\mathrm{SE}\left(\overline{\mathrm{d} }\right)=\frac{{\mathrm{SD}}_{\mathrm{d}}}{\sqrt{\mathrm{n}}}$ is the standard error of the mean difference calculated under the null hypothesis. The *P* value was considered statistically significant if less than 0.05. Bland-Altman plots, the 95% LoA, ICC of absolute agreement and the t-test were used to analyse the agreement between AL_est_ and AL_act_ in the validation dataset and OCTA dataset and agreement between factor q based on AL_est_ and AL_act_ in the OCTA dataset.

Bland-Altman plots, the 95% LoA, ICC of absolute agreement and the t-test were used to evaluate FAZA measurements after correction with AL_est_ against FAZA measurements corrected with AL_act_. The performance of AL_est_ was further evaluated by looking at how often the difference between FAZA corrected using AL_est_ and FAZA corrected using AL_act_ exceeds the coefficient of repeatability (CR). For comparison, the frequency of differences in uncorrected FAZA and FAZA corrected by AL_act_ exceeding the CR was also calculated. The frequency of exceeding the CR is calculated by |FAZA_method_ – FAZA_ALact_|> CR, where FAZA_method_ is either FAZA corrected with AL_est_ or uncorrected FAZA, and FAZA_ALact_ is FAZA corrected with AL_act_. The CR of FAZA for healthy participants imaged with the same OCTA device as in the current study has been reported as 0.052 mm^2^ (95% CI: 0.042, 0.062) mm^2^ in a study by Chen et al. [20]. The CR indicates the statistically acceptable margin of error between FAZA corrected with AL_act_ and FAZA corrected with AL_est_ or uncorrected FAZA, and thus reflects the degree to which discrepancies between AL_est_ and AL_act_ length can be tolerated without significantly affecting FAZA.

Additionally, the relative change in FAZA values corrected with AL_act_ was compared to those corrected with AL_est_ and uncorrected values. This was done with the formula 100 × (|FAZA corrected with AL_act_ − FAZA corrected with AL_est_|)/FAZA corrected with AL_act_ or 100 × (|FAZA corrected with AL_act_ − uncorrected FAZA|)/FAZA corrected with AL_act_, respectively. We also noted the maximum relative and absolute change in FAZA values corrected with AL_act_ compared to those corrected with AL_est_ and uncorrected values.

## Results

### Demographics

The demographics and ocular biometry of all study participants are summarised in Table 1; Additional file 1: Fig. S1. All study participants were phakic and had no signs of cataract formation at the time of examination.

**Table 1:** Participant demographic data and summary of ocular biometry

Parameters	Generating and validating a relationship between AL, SE, and K		Evaluating AL_est_ in correcting magnification error
	The Raine Study training dataset	The Raine Study validation dataset	OCTA test dataset
Number of participants	650	651	46
Number of eyes	650	651	46
Age (years), mean (range)	20 (19–22)	20 (18–22)	36 (23–69)
Gender (male:female)	340:310	333:318	18:28
Axial length (mm), mean (SD, range)	23.65 (0.95, 20.44–27.99)	23.58 (0.9, 20.84–26.77)	24.28 (1.38, 21.45–27.88)
Cylinder non-cycloplegic (D), mean (SD, range)	− 0.51 (0.52, − 3.50–0.00)	− 0.48 (0.47, − 4.75–0.00)	− 0.54 (0.78, − 4.00–1.25)
Cylinder post-cycloplegic (D), mean (SD, range)	− 0.52 (0.53, − 4.50–0.00)	− 0.48 (0.49, − 4.50–0.00)	NA^a^
Spherical equivalent non-cycloplegic (D), mean (SD, range)	− 0.58 (1.54, − 9.13–3.38)	− 0.51 (1.43, − 9.88–7.75)	− 1.51 (2.47, − 8.00–3.25)
Spherical equivalent post-cycloplegic (D), mean (SD, range)	− 0.15 (1.62, − 8.75–5.63)	− 0.07 (1.51, − 9.75–7.88)	NA^a^
Corneal curvature (mm), mean (SD, range)	7.76 (0.26, 7.11–8.65)	7.74 (0.26, 7.01–8.63)	7.81 (0.27, 7.24–8.64)

*AL = *axial length; *AL*_*est*_* = *estimated axial length; *D = *dioptre; *K = *keratometry; *OCTA = *optical coherence tomography angiography; *SE = *spherical equivalent; *SD = *standard deviation

^a^Post-cycloplegic SE not measured in this cohort

### Generation and validation of AL from SE and K

**Fig. 1:**
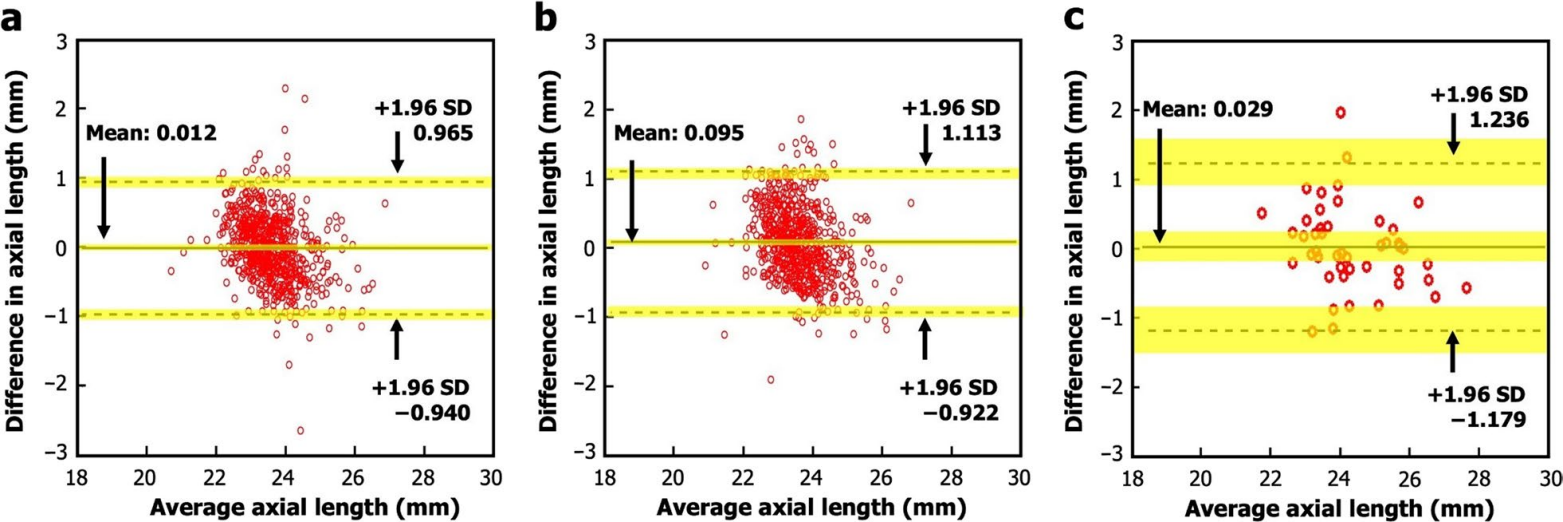
Bland-Altman plots illustrating the agreement between the AL_est_ and AL_act_ for (**a**) validation dataset (post-cycloplegic SE), (**b**) validation dataset (non-cycloplegic SE), and (**c**) OCTA dataset (non-cycloplegic SE). The yellow regions represent the 95% CI. *AL*_*act*_, actual axial length; *AL*_*est*_, estimated axial length; *CI,* confidence interval; *SE,* spherical equivalent; *SD,* standard deviation

A multiple linear regression model yielded the equation: AL_est_ = 2.102 K − 0.4125SE + 7.268; with R^2^ = 0.794. Table 2 and Fig. 1a–c show the results of the Bland-Altman and paired t-test analyses in comparing the AL_est_ and AL_act_ when applied to both the validation dataset and the OCTA test dataset. Additional file 2: Fig. S2 demonstrates the scatter plots for corresponding datasets.

**Table 2:** Bland-Altman analysis and paired t-test showing agreement between estimated and actual axial length

Parameters	Mean diff. (mm) (95% CI)	SD (mm)	Lower LoA (95% CI)	Upper LoA (95% CI)	t_stat_ (df)	*P* value	*R*^2^	ICC (95% CI)
Validation dataset (post-cycloplegic SE)	0.012 (− 0.025, 0.049)	0.486	− 0.940 (− 1.005, − 0.877)	0.965 (0.901, 1.029)	0.632 (649)	0.527	0.710	0.831 (0.806, 0.921)
Validation dataset (non-cycloplegic SE)	0.095 (0.055, 0.135)	0.519	− 0.922 (− 0.990, − 0.854)	1.113 (1.044, 1.181)	4.683 (650)	< 0.005	0.670	0.802 (0.769, 0.830)
OCTA test dataset (non-cycloplegic SE)	0.029 (− 0.154, 0.212)	0.616	− 1.179 (− 1.494, − 0.864)	1.236 (0.921, 1.551)	0.439 (45)	0.755	0.800	0.892 (0.812, 0.939)

*CI = *confidence interval; *df = *degrees of freedom; *ICC = *intraclass coefficient; *LoA = *limits of agreement; *OCTA = *optical coherence tomography angiography; *SE = *spherical equivalent; *SD = *standard deviation; *t*_*stat*_* = *t-statistic

A significant difference between AL_est_ and AL_act_ (*P* < 0.05) was noted in the non-cycloplegic validation dataset. The mean difference between AL_est_ and AL_act_ was large (0.095 mm) and LoA widths were wider compared to those of the post-cycloplegic validation dataset and non-cycloplegic OCTA test dataset. In the post-cycloplegic validation dataset and non-cycloplegic OCTA test dataset, there was no significant difference between AL_est_ and AL_act_ (*P* = 0.527 and *P* = 0.755, respectively). The mean differences between AL_est_ and AL_act_ were minimal and 95% LoA widths were narrow in both datasets. Additionally, a high ICC was reported in all datasets. Therefore, overall agreement between AL_est_ and AL_act_ in the post-cycloplegic validation dataset and OCTA test dataset can be considered as robust.

### Validation of estimated axial length on magnification error correction of FAZA

Bland-Altman analysis of factor q based on AL_est_ and AL_act_ resulted in a mean difference (SD) of 0.000 (0.008) with a 95% CI of between − 0.002 to 0.002. The 95% LoA (95% CI) were − 0.015 (− 0.020, − 0.011) and 0.016 (0.012, 0.020), the t_stat_ (df) was 0.439 (45) and *P* value was 0.754. The ICC (95% CI) was 0.892 (0.812, 0.939).

**Fig. 2:**
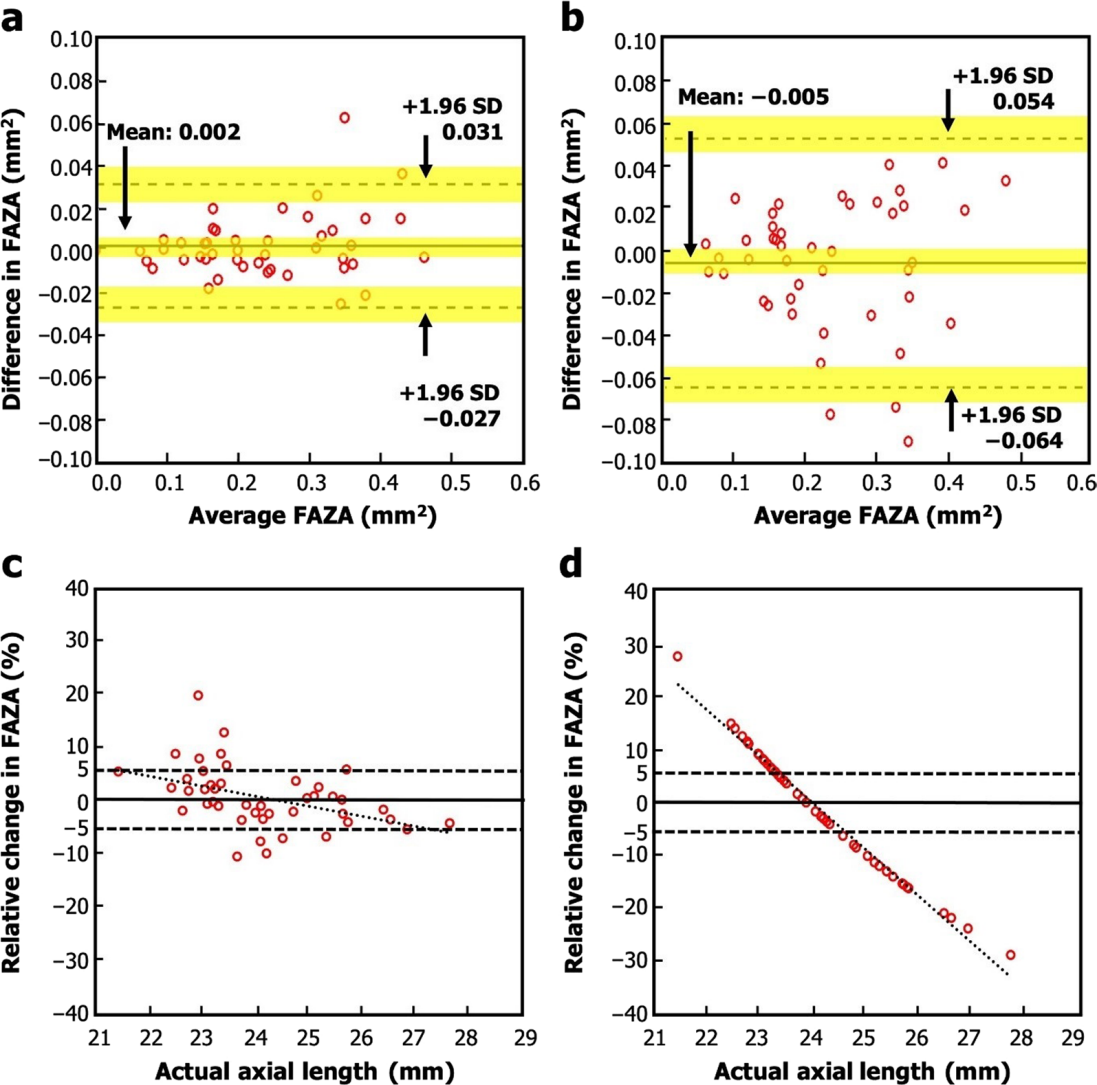
Bland-Altman plots illustrating the agreement between (**a**) FAZA corrected with AL_est_ vs. FAZA corrected with AL_act_ and (**b**) FAZA before correction vs. FAZA after correction with AL_act_. Plots of relative change between (**c**) FAZA after correction with AL_est_ vs. FAZA after correction with AL_act_, and (**d**) FAZA before correction *vs.* FAZA after correction with AL_act_. The linear dashed lines in (**c**) and (**d**) are linear fits to the data. The horizontal dashed lines in **c** and **d** indicate a 5% relative change in the FAZA. The yellow regions in **a** and **b** represent the 95% CI. *AL*_*act*_, actual axial length; *AL*_*est*_, estimated axial length; *CI,* confidence interval; *FAZA,* foveal avascular zone area; *SD,* standard deviation

The FAZA corrected for magnification error using AL_act_ were compared against both FAZA corrected using AL_est_ and uncorrected FAZA (Fig. 2; Additional file 3: Fig. S3; Table 3).

**Table 3:** Bland-Altman analysis and paired t-test showing the agreement between the FAZA measurement after correction with AL_act_ against both FAZA measurement after correction with AL_est_ and uncorrected FAZA

Parameters	Mean diff. (mm) (95% CI)	SD (mm)	Lower LoA	Upper LoA	t_stat_ (df)	*P* value	ICC (95% CI)
FAZA corrected with AL_est_ vs*.* FAZA corrected with AL_act_	0.002 (− 0.002, 0.007)	0.015	− 0.027 (− 0.034, − 0.019)	0.031 (0.024, 0.039)	1.090 (45)	0.072	0.992 (0.985, 0.995)
FAZA uncorrected vs*.* FAZA corrected with AL_act_	− 0.005 (− 0.014, 0.004)	0.030	− 0.064 (− 0.080, − 0.049)	0.054 (0.039, 0.069)	− 1.639 (45)	0.072	0.965 (0.937, 0.980)

*AL*_*act*_* = *actual axial length; *AL*_*est*_* = *estimated axial length; *CI = *confidence interval; *df = *degrees of freedom; *FAZA = *foveal avascular zone area; *ICC = *intraclass coefficient; *LoA = *limits of agreement; *SD = *standard deviation; *t*_*stat*_* = *t-statistic

The ICC and *P* value showed that FAZA corrected with AL_est_ and uncorrected FAZA did not differ significantly when compared with FAZA corrected with AL_act_. However, the lower mean difference and narrower 95% LoA indicates a stronger agreement between FAZA corrected with AL_est_ and FAZA corrected with AL_act_ compared to the agreement between uncorrected FAZA and FAZA corrected with AL_act_.

Furthermore, the frequency of uncorrected FAZA measurements exceeding the CR was 8.5%. In comparison, the frequency of exceeding the CR was reduced to 2.1% if the magnification error was corrected using the AL_est_.

A change of more than 5% in FAZA values corrected with AL_act_ compared to those corrected with AL_est_ and uncorrected vales was observed in 32% (n = 15) and 66% (n = 31) of participants, respectively. The maximum change in FAZA values corrected with AL_act_ compared to those corrected with AL_est_ and uncorrected values was 19% (0.06 mm^2^) and 28% (0.08 mm^2^), respectively.

## Discussion

The multiple linear regression model generated in this study to estimate AL, along with the application of this estimated AL in two different datasets, confirmed observations made previously that SE and K can be used to predict AL [15, 21]. Furthermore, we have shown that AL_est_ can be applied for correcting eye transverse magnification error in FAZA measurements of OCTA image. Post-cycloplegic data from the training dataset were used to generate the relationship between AL, SE, and K. Here, the use of post-cycloplegic training data may increase the accuracy of the regression formulae as cycloplegia mitigates the varying effects of tonic accommodation [22–25].

The AL_est_ formula in the present study did not include age as a variable, as population studies show that age may not have a statistically significant impact on axial length [26, 27]. Contrary to this is a study by Atchison et al. [28], which found older emmetropic eyes have longer axial lengths than younger emmetropic eyes. However, the correlation was poor (R^2^ = 0.04) and there were no significant trends for males or females alone. Atchison et al. [28] noted that this weak trend was primarily due to increases in lens thickness, with minimal changes in vitreous depth. Changes in lens thickness may contribute to changes in refractive error [26, 29], and thus will be reflected in the formula for AL_est_ in the present study. Nevertheless, the performance of AL_est_ in different age groups may be explored in the future.

Validation of AL_est_ against AL_act_ using Bland-Altmann analysis in the Raine Study Gen2-20 year follow-up cohort resulted in a lower mean difference and LoA for post-cycloplegic SE compared to non-cycloplegic SE datasets. Application of the AL_est_ formula on the post-cycloplegic data of the Raine Study Gen2-20 year follow-up cohort would appropriately produce the smallest mean difference due to the mitigation of the effects of accommodation on SE. The larger mean difference between AL_est_ and AL_act_ observed in the non-cycloplegic SE data of the Raine Study Gen2-20 year follow-up group compared to that of the OCTA cohort may be explained by the relationship between age and cycloplegia. In a younger age group, it is known that the effect of accommodation is stronger, hence cycloplegia will result in a greater reduction in SE due to reduction in myopia. The mean ages of the Raine Study Gen2-20 year follow-up cohort and the OCTA cohort were 20 and 36 years, respectively. Hence, accommodation would have a greater impact on the non-cycloplegic SE values in the Raine Study Gen2-20 year follow-up cohort compared to that of the OCTA cohort, resulting in a greater mean difference.

In the present study, both AL_est_ and AL_act_ was applied in the Littmann-Bennett formula to correct for magnification error in FAZA. It is important to note that while the Littmann-Bennett formula is treated as the gold standard in magnification error calculation, a recent study by Lal et al. [30] questions the use of the Littmann-Bennett formula in correcting OCTA image magnification error and concluded that SE should be considered in conjunction with AL when correcting OCTA image magnification factor in patients with anterior eye changes, such as in contact lens use or post refractive surgery. AL_est_ and AL_act_ can be reliably applied and compared in FAZA magnification error correction using the Littmann-Bennet formula in the present study, as all participants had healthy anterior segments. Furthermore, Lal et al. [30] corrected for magnification error of vessel density, which is less sensitive and more difficult to correct for than FAZA and benchmarked their findings against a study by Obadas et al. [31] for clinical significance. However, these two studies used different imaging and analysis protocols. Lal et al. used the Zeiss AngioPlex, Cirrus HD-OCT 5000, Carl Zeiss Meditec Inc., which defines vessel density as the total vessel length by the image size within the central 1 mm macula zone in a 3 × 3 mm acquisition area whilst Obadas et al. [31] used the Revue XR Avanti system, Optovue, which defines vessel density as the area of all white pixels within an OCTA image divided by the total image size of the whole 6 × 6 mm image. Due to the differences in methodologies, the conclusions by Lal et al. [30] cannot be applied to our study.

The mean difference of the AL_est_ values with those of AL_act_ values have been reported in previous studies. Estimations of AL by Grosvenor and Scott [32] using linear regression models with corneal radius and SE yielded a mean difference of 0.38 mm (95% LoA: − 0.60, 1.36) mm, which is higher than the mean difference found in the present study. This may be due to the inaccuracies that arise from the type of linear regression model used by Grosvenor and Scott [32], which analysed the relationship between the AL/K ratio and SE values. Kim et al. [21] estimated AL using a formula derived from the Gullstrand model of the eye, and reported a mean difference of 0.18 mm (95% LoA: − 0.75, 1.10) mm. This larger mean difference reported may be due to the inaccuracies introduced into the AL estimation formula due to the assumptions adopted with the use of a Gullstrand model eye. In a study by Morgan et al. [13], a linear regression model based on post-cycloplegic SE and K of 144 participants was also used to estimate AL values. This model showed a mean difference of 0.13 mm (95% LoA: − 0.73, 0.99) mm when applied to a separate validation cohort of 1046 participants aged between 6 to 22 years. This mean difference is higher but comparable to that reported in this study and may be so due to the generation of a linear regression from a small cohort.

Others have utilised semi-automated computer processing to facilitate AL estimation. Tang et al. [33] compared machine learning with traditional multiple regression formulae in developing a method for estimating AL. Regression models were based on age, gender, K, SE and white-to-white diameter. Tang et al. [33] found that machine-learning methods outperformed traditional multiple regressions model for estimating AL, with the strongest machine-learning model AL prediction model having a R^2^ of 0.86, which is considerably robust, however Tang et al. [33] did not report a mean difference or 95% LoA. A deep-learning algorithm applied to colour fundus photographs was used by Dong et al. [34] to estimate AL, and resulted in R^2^ = 0.59 and mean difference of 0.16 mm (95% LoA: − 0.60, 1.27) mm. This is a higher mean difference and wider 95% LoA than observed in the present study. AL estimation using deep neural network visual interpretation of fundus images by Jeong et al. [35] yielded R^2^ = 0.67 while the mean difference and 95% LoA were not reported. The R^2^ value of AL estimation in the present study is higher than in the estimation methods of Dong et al. [34] and Jeong et al. [35]. Hence, even though these studies are promising for the use of semi-automated estimation of AL, further research in this area is needed to improve on these methods.

Whilst semi-automated computer processing may aid in AL estimation, there is value in traditional estimation techniques such as those described in this study owing to its ease of use. This does not discount the accuracy of the AL_est_. In comparisons of the magnification error correction performance of AL_est_ with the AL estimation methods presented in the aforementioned studies [13, 21, 32–35], it is important to note that even marginal improvements in the accuracy of estimation methods may provide significant advances in magnification error correction for FAZA, and possibly for other fundus parameters like vessel density.

The AL_est_ demonstrated in the present study has robust agreement with a high ICC and narrow LoAs when compared against AL_act_ in correcting transverse magnification error. Since the work published by Sampson et al. [3], multiple studies have acknowledged the inaccuracy in fundus analysis without correcting for magnification error and have proceeded to include AL-based correction of fundus parameter analyses [20, 36, 37]. This is particularly true for FAZA measurements, especially in high myopes [38, 39]. In addition to FAZA correction, magnification error correction has been used in studies investigating macular vessel density, impacting on the accuracy of these measurements [40].

Here, FAZA values corrected with AL_act_ were not significantly different from uncorrected FAZA values or FAZA corrected with AL_est_ (*P* > 0.05, Table 3). Sampson et al. [3] also did not find a significant difference between corrected and uncorrected FAZA measurements with sample size of 67 eyes. However, the authors demonstrated that not correcting for magnification factor contributed to an error of FAZA measurements of more than 5% for 74% of their participants. This is similar to the error found in the OCTA participants of the present study when comparing uncorrected FAZA with FAZA corrected with AL_est_ and AL_act_. FAZA correction with AL also did not reach significance in a study be Linderman et al. [8] with a sample of the 232 eyes. However, Linderman et al. [8] pointed out that not correcting FAZA for the magnification factor resulted in errors to FAZA of up to 31% (0.07 mm^2^). This is comparable to the percentage changes in FAZA in OCTA participants when comparing uncorrected FAZA with FAZA corrected with AL_est_ and AL_act_.

In line with these studies, whilst FAZA correction for magnification error did not reach statistical significance, there was an important increase in the accuracy of FAZA measurements after correction for eye magnification factor. As seen in Fig. 2c, d, the deviation from the true FAZA value is higher for uncorrected FAZA than FAZA corrected with AL_est_. Therefore, FAZA corrected by the AL_est_ is more accurate than uncorrected FAZA. This is confirmed when adjusting FAZA for magnification error with AL_est_ resulted in a twofold reduction in the width of the 95% LoA, and a lower frequency of exceeding the CR in the present study, compared to uncorrected FAZA. This increase in accuracy is important for standardising FAZA measurements to enable comparisons of FAZA across studies. It may also provide more sensitive monitoring of FAZA in participants over time. Understanding this importance, in cases where AL_act_ is not available for FAZA correction, it is advisable to correct for magnification error in FAZA measurements using AL_est_ rather than accepting uncorrected measurements.

The use of AL_est_ in magnification error correction may be important in assessing the presence of disease or for monitoring disease progression in cases where actual AL measurements are unavailable. Currently, magnification factor correction is being performed more commonly when monitoring fundus parameters in diseased states such as diabetic retinopathy and diabetic macular oedema [41]. Traditionally, magnification factor correction is performed with AL_act_ measured with an ocular biometer. These devices have become increasingly popular in clinical practice and are necessary in managing myopia, in conjunction with routine corneal parameters. However, there may be instances where retrospective correction of magnification error in OCTA images is needed to be performed in the absence of axial length measurements; our study allows for this if K and SE are known.

In clinics without access to these biometry devices, the present study suggests that AL_est_ may have a role in correcting magnification factor for monitoring fundus parameters in diseases that impact the fundus. The use of AL_est_ may also provide for a more cost-effective means of performing magnification factor correction than using AL_act_, as specialised biometry devices are not required. Future studies should assess the performance of AL_est_ in magnification error correction in a range of diseased states including diabetic retinopathy, age related macular degeneration and glaucoma. Additionally, conditions that impact the anterior segment of the eye, such as keratoconus, cataracts, and refractive surgery, may result in changes to refractive error and K results independent of AL [30], and thus the performance of AL_est_ in magnification error correction in these conditions should also be studied.

Future studies may also wish to assess the performance of AL_est_ in myopic or hyperopic cohorts specifically, as the training, validation and test cohorts of the present study include participants with a wide range of refractive errors. In addition, FAZA magnification error correction with AL_est_ resulted in an overestimation of FAZA for shorter AL and an underestimation of FAZA for longer AL, when compared to magnification error correction of FAZA with AL_act_ (Fig. 2c; Additional file 4: Fig. S4). This may indicate the need for further refinement of the AL_est_ formula in myopic or hyperopic conditions. However, clinicians should be reassured that many of these differences in FAZA corrected with AL_est_ and AL_act_ are subtle, with 98.9% of measurements falling within the CR. Rates of myopia may vary between ethnicities in paediatric populations [42, 43]. However, the impact of ethnicity on rates of myopia in adults is less clear [44]. Nevertheless, the Raine Study Gen2-20 year follow-up cohort is predominately Caucasian, and hence the performance of AL_est_ in cohorts of different ethnic backgrounds will be an opportunity for further study.

The instruments used to determine K and autorefraction in the training and validation cohorts were different from those of the test cohort. Since measurements from different machines may vary for a given parameter, future studies may test the performance of AL_est_ across different instrumentations and measurement protocols. Additionally, future studies may choose to use larger ranges of AL, K and SE to assess if differences between uncorrected FAZA and FAZA corrected with AL_est_ or AL_act_ reach statistical significance and is of clinical relevance. It is important to note that though FAZA has a strong dependence on magnification error correction, the effect of image correction also impacts vessel density. Vessel density magnification error correction is also influenced by the FAZA included in the region of analysis. To truly determine the effectiveness of the proposed formula for AL_est_, future studies should expand the use of AL_est_ in assessing the accuracy in vessel density magnification error correction.

## Conclusion

Our data suggest that AL_act_ and AL_est_ are comparable, especially when post-cycloplegic SE is used. However, when applying AL in magnification error correction, AL_act_ is more accurate than AL_est_ and should be used preferentially in the clinical setting. In the absence of AL_act_, clinicians should avoid relying on uncorrected measurements, and instead should use AL_est_ to correct for magnification error as this provides for a more accurate measurement of fundus parameters.

## Supplementary Information

**Additional file 1: Fig S1.** Distribution of the axial length (AL), spherical equivalent (SE) and keratometry in **(a)** training dataset (post-cycloplegic SE), **(b)** validation dataset (post-cycloplegic SE), and **(c)** OCTA dataset (non-cycloplegic SE).

**Additional file 2: Fig S2.** Scatter plots between the AL_est_ and AL_act_ for **(a)** validation dataset (post-cycloplegic SE), **(b)** validation dataset (non-cycloplegic SE), and **(c)** OCTA dataset (non-cycloplegic SE). AL_act_, actual axial length; AL_est_, estimated axial lengt; CI, confidence interval; SE, spherical equivalent.

**Additional file 3: Fig S3.** Scatter plots between **(a)** FAZA corrected with AL_est_ and AL_act_ **(b)** FAZA before correction and FAZA corrected with AL_act_. AL_act_, actual axial length; AL_est_, estimated axial length; FAZA, foveal avascular zone area.

**Additional file 4: Fig S4.** Impact of correcting transverse magnification error in OCTA using AL_act_ and AL_est_ on OCTA image size and FAZA measurement. Correction of the OCTA image for the study participant with **(a)** a shorter eye in our cohort (AL = 21.45 mm) and **(b)** a longer eye (AL = 27.88 mm). AL_act_, actual axial length; AL_est_, estimated axial length; FAZA, foveal avascular zone area; OCTA, optical coherence tomography angiography.

## Data Availability

The Raine Study Gen2-20 year follow-up data that support the findings of this study are available from The Raine Foundation, but restrictions apply to the availability of these data, which were used under license for the current study, and so are not publicly available. Data are however available from the authors upon reasonable request and with permission of The Raine Foundation.

## Abbreviations

AL_act_: Actual axial length
AL_est_: Estimated axial length
CR: Coefficient of repeatability
FAZA: Foveal avascular zone area
ICC: Intraclass coefficient
K: Keratometry
LoA: Limits of agreement
OCTA: Optical coherence tomography angiography
q: Magnification factor of the eye
SE: Spherical equivalent
SD: Standard deviation
t_stat_: T-statistic
