# Supplementary figures and images for: Correcting magnification error in foveal avascular zone area measurements of optical coherence tomography angiography images with estimated axial length
Source: Eye Vis (Lond). 2022 Aug 1;9:29. doi: 10.1186/s40662-022-00299-x (PMC9341098; doi:10.1186/s40662-022-00299-x)

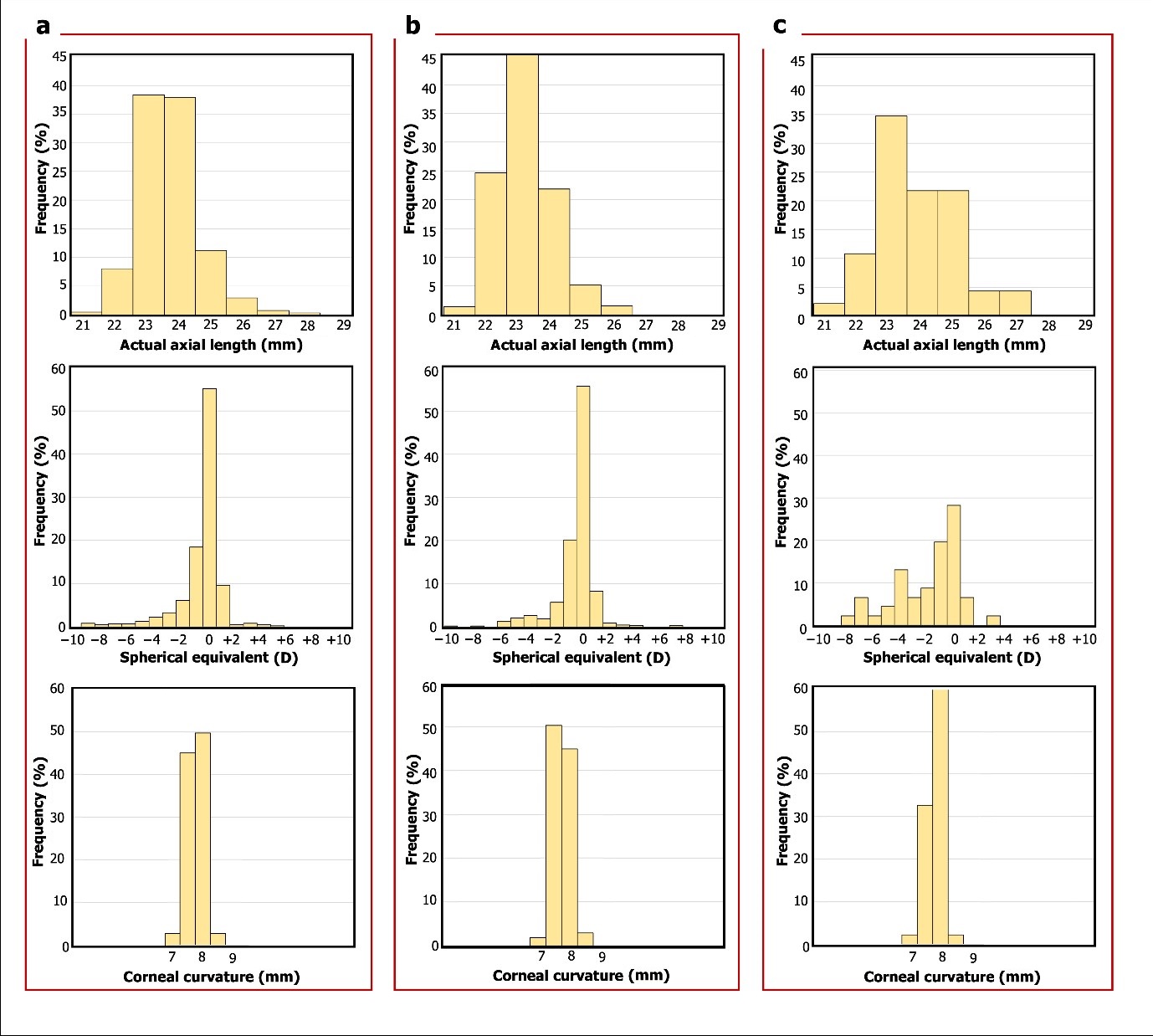

Supplement: Supplementary file 1 — Additional file 1: Fig S1. Distribution of the axial length (AL), spherical equivalent (SE) and keratometry in (a) training dataset (post-cycloplegic SE), (b) validation dataset (post-cycloplegic SE), and (c) OCTA dataset (non-cycloplegic SE). [file 40662_2022_299_MOESM1_ESM.jpg]

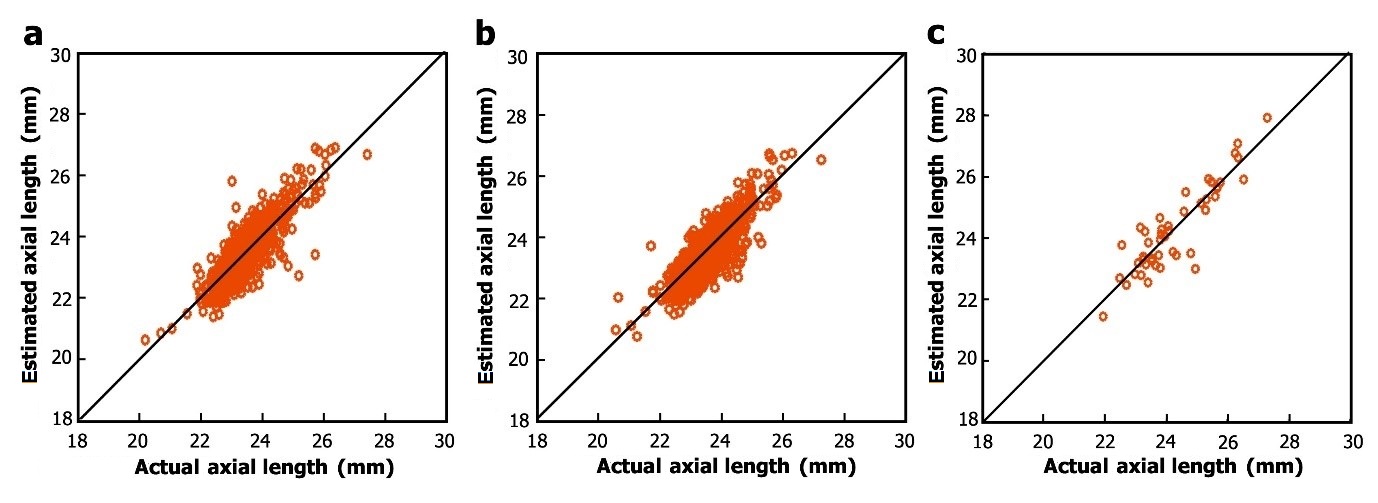

Supplement: Supplementary file 2 — Additional file 2: Fig S2. Scatter plots between the ALest and ALact for (a) validation dataset (post-cycloplegic SE), (b) validation dataset (non-cycloplegic SE), and (c) OCTA dataset (non-cycloplegic SE). ALact, actual axial length; ALest, estimated axial lengt; CI, confidence interval; SE, spherical equivalent. [file 40662_2022_299_MOESM2_ESM.jpg]

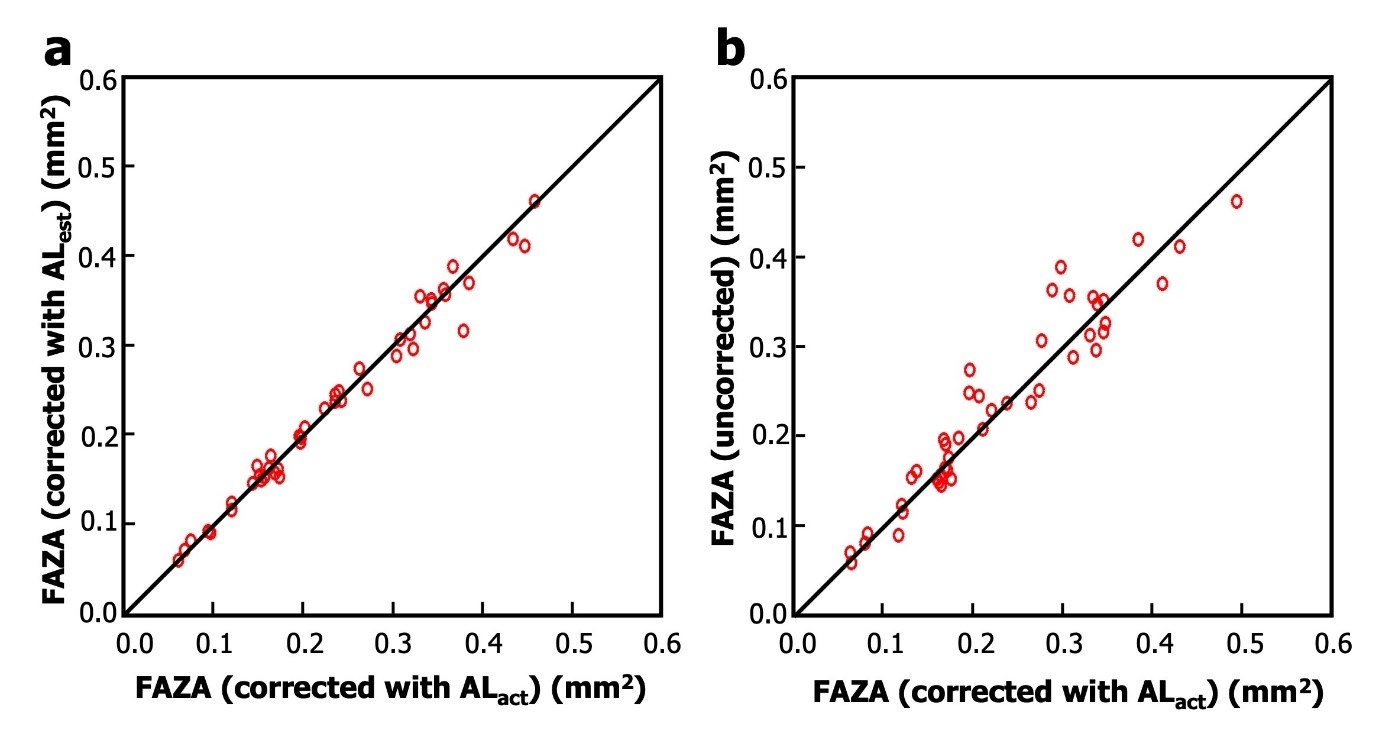

Supplement: Supplementary file 3 — Additional file 3: Fig S3. Scatter plots between (a) FAZA corrected with ALest and ALact (b) FAZA before correction and FAZA corrected with ALact. ALact, actual axial length; ALest, estimated axial length; FAZA, foveal avascular zone area. [file 40662_2022_299_MOESM3_ESM.jpg]

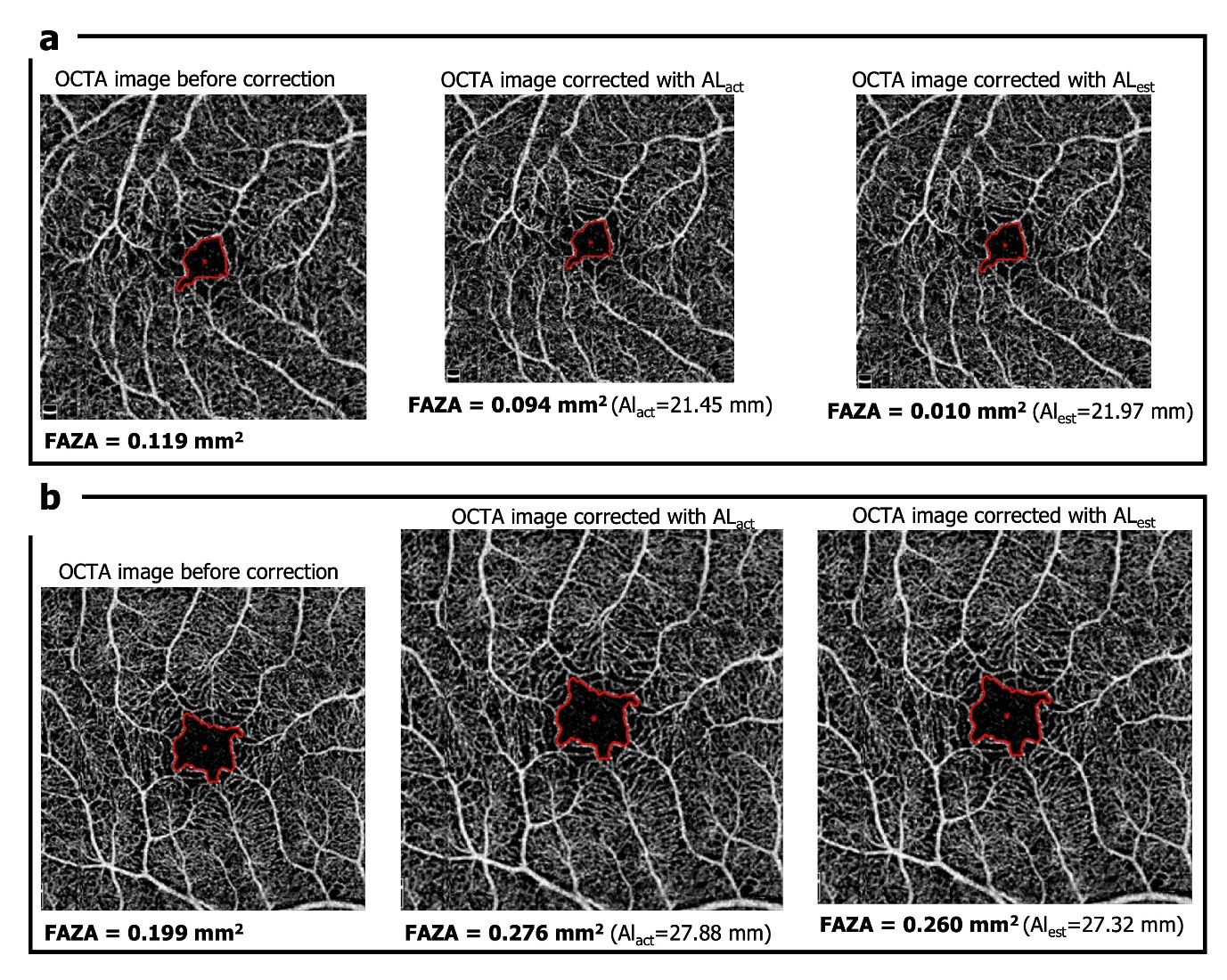

Supplement: Supplementary file 4 — Additional file 4: Fig S4. Impact of correcting transverse magnification error in OCTA using ALact and ALest on OCTA image size and FAZA measurement. Correction of the OCTA image for the study participant with (a) a shorter eye in our cohort (AL = 21.45 mm) and (b) a longer eye (AL = 27.88 mm). ALact, actual axial length; ALest, estimated axial length; FAZA, foveal avascular zone area; OCTA, optical coherence tomography angiography. [file 40662_2022_299_MOESM4_ESM.jpg]
